# Data on long noncoding RNA upregulated in hypothermia treated cardiomyocytes protects against myocardial infarction through improving mitochondrial function

**DOI:** 10.1016/j.dib.2018.01.052

**Published:** 2018-02-05

**Authors:** Jian Zhang, Liming Yu, Yinli Xu, Yu Liu, Zhi Li, Xiaodong Xue, Song Wan, Huishan Wang

**Affiliations:** a Department of Cardiovascular Surgery, General Hospital of Shenyang Military Area Command, No.83, Wenhua Road, Shenhe District, Shenyang, Liaoning 11001, China; b Department of Cardiothoracic Surgery, The Chinese University of Hong Kong, Prince of Wales Hospital, Shatin, NT, Hong Kong

## Abstract

This article elaborates on cardioprotective action of hypothermia related long noncoding RNA against myocardial infarction through improving mitochondrial function, which preset by J Zhang. Herein, we provide the materials and methods used in that study. And provided the detail of dysregulation of lncRNAs under the treatment of hypothermia. Furthermore, we found that lnc-UIHTC (lncRNA upregulated in hypothermia treated cardiomyocyte, NONHSAT094064) attenuated cardiomyocytes apoptosis *in vitro.*

**Specifications Table**

**Table:** 

Subject area	*Biology*
More specific subject area	*Hypothermia and cardiology*
Type of data	*Tables and figures*
How data was acquired	*Seahorse XF96 extracellular flux analyzer.*
Data format	*Analyzed*
Experimental factors	*Adult ventricular cardiomyocyte cells (AC16) was treated with hypothermia and transfected with UIHTC.*
Experimental features	*Cells were cultured at 37 °C or 28 °C with 5% CO2 for 6 h*
Data source location	*Shenyang city, Liaoning province, China*
Data accessibility	*Data are presented in this article*

**Value of the data**

• The data provides overexpression of UIHTC inhibited H_2_O_2_-induced AC16 apoptosis.
• This data provides the details of differentially expressed lncRNAs of cardiomyocytes exposed on hypothermia.
• The data may stimulate further research on the function of lncRNAs stimulated in cardiomyocytes under hypothermia.

## 1 Data

The details of changed lncRNAs were listed in Table 1 (Table 1).

**Table 1:** Details of changed lncRNAs in hypothermia treated AC16 cells.

**Genbank accession**	**Regulation**	**Chr**	**Chr strand**	**Fold change**
n4541	Up	chr22	+	2.16
n342704	Up	chr17	−	2.66
NR_002983	Up	chr1	−	1.62
n334074	Up	chr17	−	2.18
n339087	Up	chr2	+	1.59
TCONS_00011996-XLOC_005510	Up	chr6	+	1.89
n408293	Up	chr17	−	1.64
n333380	Up	chr3	+	1.98
ENST00000365207	Up	chr17	−	1.63
n334398	Up	chr17	+	1.81
NR_034120	Up	chr7	−	2.05
TCONS_00025394-XLOC_012209	Up	chr17	+	1.77
n340430	Up	chr5	−	1.68
n337368	Up	chr19	+	1.91
NR_026582	Up	chr3	−	1.64
ENST00000364127	Up	chr17	−	2.07
ENST00000273411	Up	chr3	−	1.72
n333278	Up	chr12	−	1.89
n407948	Up	chr10	−	1.74
n339176	Up	chr2	−	1.81
ENST00000384476	Up	chr1	−	1.57
n342580	Up	chr18	+	1.98
TCONS_00019243-XLOC_009074	Up	chr11	+	1.44
n378377	Up	chr7	−	1.85
n333449	Up	chr6	+	1.36
NR_002921	Up	chr2	−	1.69
NR_002581	Up	chr8	−	1.57
ENST00000365538	Up	chr1	−	1.95
ENST00000385251	Up	chr16	+	1.42
n340431	Up	chr5	−	1.49
n342718	Up	chr6	+	1.61
n5726	Up	chr15	−	1.68
n334819	Up	chr10	+	1.56
n387280	Up	chr3	−	1.55
ENST00000532242	Up	chr11	+	1.36
ENST00000364829	Up	chr1	+	1.63
ENST00000499503	Up	chr17	+	1.34
ENST00000384770	Up	chr1	+	1.4
ENST00000384241	Up	chr1	−	1.43
ENST00000516747	Up	chr11	+	1.37
ENST00000390893	Up	chr17	−	1.66
n337074	Up	chr2	+	1.41
n335609	Up	chr17	+	1.52
n337037	Up	chr21	−	1.34
ENST00000364166	Up	chr14	+	1.45
n334787	Up	chr4	+	2.68
n386326	Up	chr3	+	1.38
ENST00000442269	Up	chr6	+	1.68
n341319	Up	chr7	−	1.35
TCONS_00016056-XLOC_007446	Up	chr9	+	1.36
NR_003199	Up	chr14	+	1.41
n335550	Up	chr14	−	1.32
n339341	Up	chr3	+	1.59
ENST00000550319	Up	chr12	+	1.37
TCONS_00003553-XLOC_001325	Up	chr2	+	1.35
n336002	Up	chr8	−	2.43
ENST00000425077	Up	chr7	−	1.37
n334456	Up	chr5	−	1.45
ENST00000391122	Up	chr15	+	1.4
n340425	Up	chr5	+	1.48
TCONS_00029913-XLOC_014386	Up	chr22	−	1.37
NR_024333	Up	chr19	−	1.41
n323941	Up	chr9	+	1.32
ENST00000391154	Up	chr1	+	1.46
n339466	Up	chr21	−	1.35
NR_002971	Up	chr6	+	1.7
NR_002971	Up	chr6_cox_hap2	+	1.7
NR_002971	Up	chr6_mann_hap4	+	1.7
NR_002971	Up	chr6_qbl_hap6	+	1.7
NR_002971	Up	chr6_ssto_hap7	+	1.7
n338881	Up	chr2	−	1.59
OTTHUMT00000035200	Up	chr6_qbl_hap6	−	1.33
n334500	Up	chr14	+	1.47
n339072	Up	chr2	−	1.34
n340075	Up	chr10	−	1.44
ENST00000384746	Up	chr12	+	1.32
ENST00000391248	Up	chr15	−	1.36
n379365	Up	chr17	+	1.31
ENST00000462262	Up	chr21	+	1.38
n378747	Up	chr18	+	1.34
n333545	Up	chr19	+	1.54
n408024	Up	chrX	+	1.53
ENST00000384700	Up	chr6	−	1.38
n339593	Up	chr20	+	1.34
TCONS_00019032-XLOC_008957	Up	chr10	−−	1.62
ENST00000363009	Up	chr1	+	1.43
TCONS_00002160-XLOC_000401	Up	chr1	+	1.34
ENST00000428367	Up	chr17	−	1.36
n406399	Up	chr7	−	1.33
ENST00000425678	Up	chr2	−	1.31
n334125	Up	chr7	+	1.33
n340509	Up	chr5	−	1.4
n340071	Up	chr10	+	1.37
n342795	Up	chr7	+	1.34
n338895	Up	chr2	+	1.37
n340154	Up	chr10	−	1.59
n326361	Up	chr1	−	1.32
ENST00000410792	Up	chr2	−	1.37
TCONS_l2_00005211-XLOC_l2_002790	Up	chr11	−	1.54
n334591	Up	chr11	+	1.35
NR_002956	Up	chr1	−	1.34
n409625	Up	chr20	+	1.31
n407093	Up	chr20	+	1.32
ENST00000383967	Up	chr1	+	1.62
ENST00000364313	Up	chr1	+	1.62
TCONS_00014808-XLOC_006883	Up	chr8	+	1.35
NR_037869	Up	chr1	−	1.34
n377834	Up	chr5	+	1.35
TCONS_00024489-XLOC_011809	Up	chr16	+	1.31
ENST00000447372	Up	chr22	−	1.31
n341643	Up	chr9	+	1.31
ENST00000537889	Up	chr16	+	1.32
TCONS_00024338-XLOC_011654	Up	chr16	+	1.39
NR_003210	Up	chr14	+	1.4
n339184	Up	chr2	+	1.32
ENST00000384367	Up	chr1	+	1.38
n380676	Up	chr11	−	1.42
ENST00000515085	Up	chr5	+	1.38
n332643	Up	chr10	+	1.41
ENST00000384601	Up	chr3	+	1.53
n336606	Up	chr11	−	1.35
ENST00000538067	Up	chr12	+	1.33
TCONS_00006915-XLOC_002730	Up	chr3	+	1.49
n340869	Up	chr6	+	1.88
TCONS_00018082-XLOC_008337	Up	chr10	+	1.4
ENST00000449168	Up	chr2	+	1.4
ENST00000516287	Up	chr20	+	1.38
TCONS_00019651-XLOC_009456	Up	chr11	−	1.32
ENST00000416061	Up	chrX	−	1.32
n334635	Up	chr5	+	1.39
n410630	Up	chr1	−	1.46
ENST00000411067	Up	chr18	−	1.32
TCONS_00010299-XLOC_004777	Up	chr5	−	1.34
NR_001285	Up	chr19	+	1.31
TCONS_00009322-XLOC_004238	Up	chr5	+	1.39
ENST00000383927	Up	chr1	−	1.35
ENST00000414515	Up	chr9	−	1.41
n345970	Up	chr2	+	1.42
ENST00000516357	Up	chrY	+	1.33
ENST00000516704	Up	chrY	−	1.33
n341216	Up	chr7	+	1.38
ENST00000456346	Up	chr6	−	1.43
ENST00000410361	Up	chr9	−	1.31
TCONS_00002934-XLOC_001531	Up	chr2	+	1.32
TCONS_l2_00030929-XLOC_l2_015938	Up	chrY	−	1.38
ENST00000384265	Up	chr12	+	1.34
TCONS_00011337-XLOC_005383	Up	chr6	+	1.35
TCONS_00004043-XLOC_001882	Up	chr2	+	1.36
TCONS_00000574-XLOC_000920	Up	chr1	−	1.34
n332690	Up	chr20	−	1.31
ENST00000432268	Up	chr2	−	1.35
n386063	Up	chr14	−	1.36
ENST00000363626	Up	chr11	+	1.32
n346101	Up	chrX	+	1.59
ENST00000384371	Up	chr12	+	1.32
n341052	Up	chr11	+	1.32
ENST00000516697	Up	chr1	+	1.31
ENST00000557144	Up	chr4	−	1.33
ENST00000384032	Up	chr3	−	1.33
TCONS_00019540-XLOC_009333	Up	chr11	+	1.31
TCONS_00000713-XLOC_001151	Up	chr1	−	1.36
ENST00000364243	Up	chr2	−	1.33
n378384	Up	chr2	−	1.53
ENST00000384246	Up	chr2	+	1.34
ENST00000441217	Up	chr2	+	1.35
n334057	Up	chr5	−	1.32
ENST00000411281	Up	chr12	+	1.51
NR_038970	Up	chr14	−	1.34
n406921	Down	chr2	+	0.23
n409260	Down	chr3	+	0.41
n345255	Down	chr14	+	0.47
ENST00000549251	Down	chr12	−	0.48
n342393	Down	chr19	+	0.43
n340899	Down	chr6	+	0.54
n339467	Down	chr3	+	0.53
n410890	Down	chr5	+	0.61
n342817	Down	chr7	+	0.52
n407477	Down	chr3	+	0.53
n340730	Down	chr5	−	0.52
n334786	Down	chr10	−	0.49
NR_024214	Down	chr19	+	0.58
NR_004435	Down	chr19	+	0.58
TCONS_l2_00022861-XLOC_l2_012011	Down	chr5	−	0.56
n410211	Down	chr1	+	0.59
n385685	Down	chr9	−	0.4
ENST00000375210	Down	chr9	−	0.51
n407522	Down	chr1	+	0.66
n338319	Down	chr14	+	0.57
NR_003075	Down	chr7	+	0.56
n384655	Down	chr5	−	0.64
n337872	Down	chr1	−	0.54
n408031	Down	chr3	−	0.64
n340901	Down	chr6	+	0.55
n340647	Down	chr5	+	0.54
n384393	Down	chr3	−	0.64
n340510	Down	chr17	+	0.48
NR_004437	Down	chr19	+	0.6
NR_002951	Down	chr12	−	0.65
n342113	Down	chr6	−	0.65
n341217	Down	chr7	+	0.6
n335577	Down	chr4	+	0.59
n384996	Down	chr6	+	0.6
n383211	Down	chr17	−	0.67
n408051	Down	chr2	−	0.49
n410523	Down	chr5	−	0.58
n345681	Down	chr1	−	0.68
n335651	Down	chr22	−	0.61
n332602	Down	chr1	+	0.52
n409777	Down	chr10	+	0.62
n407842	Down	chr1	+	0.56
n405416	Down	chr19	+	0.71
NR_003016	Down	chr4	+	0.52
n338183	Down	chr1	−	0.52
n386362	Down	chr4	+	0.5
n342253	Down	chr1	+	0.67
n341520	Down	chr8	−	0.61
n341502	Down	chr8	−	0.68
ENST00000408820	Down	chr4	+	0.63
ENST00000408155	Down	chr4	−	0.63
n385279	Down	chr7	+	0.7
n340607	Down	chr5	+	0.66
TCONS_l2_00010617-XLOC_l2_005701	Down	chr17	+	0.71
NR_026705	Down	chr5	+	0.57
n342192	Down	chr3	+	0.65
n341987	Down	chr12	−	0.58
ENST00000459523	Down	chr14	+	0.5
TCONS_00029063-XLOC_013984	Down	chr21	+	0.68
n409199	Down	chr3	−	0.75
NR_028308	Down	chr2	−	0.74
n383019	Down	chr16	+	0.75
ENST00000408329	Down	chr4	−	0.52
ENST00000408285	Down	chr4	−	0.52
NR_003608	Down	chr22	−	0.76
n342056	Down	chr12	+	0.65
n338696	Down	chr19	+	0.59
TCONS_00007758-XLOC_003943	Down	chr4	−	0.66
n407180	Down	chr17	−	0.66
n407319	Down	chr3	+	0.75
n386478	Down	chr8	−	0.65
n346330	Down	chr4	+	0.66
n342890	Down	chr12	+	0.56
n340068	Down	chr10	−	0.68
n382189	Down	chr14	−	0.51
TCONS_00001109-XLOC_000350	Down	chr1	+	0.67
NR_037803	Down	chr11	+	0.68
n336825	Down	chr8	+	0.75
n332871	Down	chr7	+	0.76
n383770	Down	chr2	−	0.44
n337209	Down	chr10	+	0.73
NR_002922	Down	chr5	+	0.71
NR_002576	Down	chr17	−	0.75
n410156	Down	chr3	+	0.73
n405970	Down	chr10	+	0.69
n335076	Down	chr12	+	0.72
NR_002995	Down	chr17	+	0.66
ENST00000446984	Down	chr9	−	0.76
n385776	Down	chrX	+	0.7
n408092	Down	chr11	−	0.62
n335676	Down	chr4	−	0.62
ENST00000459390	Down	chr1	+	0.39
ENST00000458828	Down	chr1	−	0.39
n385638	Down	chr9	−	0.66
TCONS_00004331-XLOC_002204	Down	chr2	−	0.63
ENST00000517046	Down	chrY	−	0.56
ENST00000459234	Down	chr2	+	0.59
ENST00000411167	Down	chr4	−	0.63
n406823	Down	chr12	+	0.73
ENST00000408237	Down	chr10	−	0.71
n344778	Down	chr15	+	0.67
n338270	Down	chr19	−	0.69
n335672	Down	chr7	+	0.6
n332583	Down	chr10	−	0.7
n335635	Down	chr1	+	0.69
n333316	Down	chr19	+	0.69
n408238	Down	chr20	+	0.75
NR_003059	Down	chr16	−	0.72
TCONS_00000861-XLOC_000086	Down	chr1	+	0.67
n410735	Down	chr12	−	0.7
NR_024221	Down	chr19	+	0.67
ENST00000459294	Down	chr1	−	0.53
ENST00000553825	Down	chr14	+	0.7
n333463	Down	chr5	+	0.71
n380727	Down	chr11	+	0.74
n410524	Down	chr1	−	0.71
n385631	Down	chr9	+	0.73
n384424	Down	chr4	+	0.63
TCONS_00008434-XLOC_003881	Down	chr4	−	0.76
n410120	Down	chr5	−	0.7
NR_024218	Down	chr19	+	0.65
n341422	Down	chr13	+	0.7
n335490	Down	chr5	−	0.61
n409529	Down	chr2	−	0.65
n340203	Down	chr4	+	0.76
TCONS_l2_00017541-XLOC_l2_009280	Down	chr22	+	0.65
n332758	Down	chr19	−	0.6
n386687	Down	chr11	+	0.71
n338700	Down	chr2	−	0.72
n334497	Down	chr12	+	0.68
n335646	Down	chr20	−	0.54
n385468	Down	chr8	−	0.73
ENST00000517174	Down	chr7	+	0.64
TCONS_l2_00019469-XLOC_l2_010312	Down	chr3	−	0.5
TCONS_l2_00013033-XLOC_l2_007013	Down	chr19	−	0.7
ENST00000459307	Down	chr21	−	0.45
n337011	Down	chr15	+	0.67
ENST00000410505	Down	chr4	+	0.52
TCONS_00025203-XLOC_012351	Down	chr17	+	0.68
n338975	Down	chr2	−	0.61
n338468	Down	chr14	−	0.76
n384600	Down	chr5	+	0.65
ENST00000425109	Down	chr1	−	0.71
n384667	Down	chr5	−	0.71
n332927	Down	chrX	+	0.76
ENST00000381105	Down	chrX	−	0.71
n338489	Down	chr1	−	0.58
n339117	Down	chr2	+	0.74
NR_021492	Down	chr22	+	0.62
n386409	Down	chr6	+	0.64
ENST00000517242	Down	chr13	−	0.75
NR_026757	Down	chr15	+	0.67
n406648	Down	chr4	+	0.74
ENST00000503553	Down	chr5	−	0.68
TCONS_l2_00002830-XLOC_l2_001417	Down	chr1	−	0.65
n383778	Down	chr2	+	0.74
NR_033931	Down	chr4	+	0.73
n341339	Down	chr7	−	0.69
n338835	Down	chr12	−	0.65
n341945	Down	chr12	+	0.7
n341914	Down	chr12	−	0.55
n332774	Down	chr11	+	0.76
n342272	Down	chr22	−	0.7
n338494	Down	chr14	+	0.72
ENST00000458806	Down	chr1	−	0.76
n410543	Down	chr2	−	0.69
n342223	Down	chr1	+	0.63
ENST00000417820	Down	chr21	+	0.69
n410892	Down	chr14	+	0.75
NR_003010	Down	chr12	−	0.7
n409093	Down	chr12	+	0.64
TCONS_00003380-XLOC_002282	Down	chr2	−	0.67
NR_002980	Down	chr16	−	0.68
n332620	Down	chr1	+	0.62
n410123	Down	chr22	−	0.73
n325691	Down	chr13	+	0.73
ENST00000363272	Down	chr15	−	0.69
n345178	Down	chr12	+	0.71
n339370	Down	chr3	−	0.69
n410169	Down	chr9	+	0.75
ENST00000516845	Down	chr20	+	0.74
NR_023392	Down	chr8	−	0.73
n337863	Down	chr1	+	0.73
ENST00000459322	Down	chr9	−	0.44
ENST00000499250	Down	chr8	−	0.76
n410486	Down	chr20	+	0.66
n345222	Down	chr13	+	0.71
n337929	Down	chr1	−	0.65
ENST00000362760	Down	chr6	+	0.63
n341238	Down	chr7	−	0.72
ENST00000459317	Down	chr9	−	0.48
n344934	Down	chr6	−	0.72
n335516	Down	chr17	−	0.72
n341846	Down	chr6	−	0.61
n381331	Down	chr1	−	0.6
TCONS_00023251-XLOC_011554	Down	chr15	−	0.71
n340611	Down	chr5	+	0.64
NR_034144	Down	chr17	+	0.64
n333320	Down	chr1	+	0.72
n342611	Down	chr4	+	0.73
ENST00000435109	Down	chr2	−	0.71
n332938	Down	chr2	−	0.75
n332918	Down	chr1	+	0.75
n3893	Down	chr12	−	0.7
n408049	Down	chr2	+	0.75
n407172	Down	chr5	−	0.74
NR_002977	Down	chr11	+	0.76
n342784	Down	chr11	−	0.66
ENST00000429933	Down	chr13	+	0.75
n406963	Down	chr3	+	0.71
n387024	Down	chr17	−	0.66
ENST00000459169	Down	chr21	+	0.75
n339642	Down	chr3	+	0.75
ENST00000410413	Down	chr3	+	0.72
NR_004387	Down	chr12	+	0.73
n333096	Down	chr1	+	0.73
ENST00000408488	Down	chr4	+	0.41
n384454	Down	chr4	−	0.61
n335600	Down	chr4	+	0.71
n339419	Down	chr3	+	0.7
ENST00000458847	Down	chr21	−	0.4
n406459	Down	chr3	−	0.76
n338422	Down	chr14	−	0.69
n340381	Down	chr5	+	0.73
ENST00000553202	Down	chr12	−	0.69
n340150	Down	chr10	−	0.76
n337962	Down	chr1	−	0.74
NR_046097	Down	chr1	+	0.76
ENST00000459257	Down	chr1	+	0.48
n336936	Down	chr10	−	0.75
n335470	Down	chr6	+	0.72
n342582	Down	chr18	+	0.7
TCONS_l2_00020697-XLOC_l2_010802	Down	chr4	+	0.71
n339794	Down	chr18	−	0.76
n342579	Down	chr4	+	0.76
ENST00000390127	Down	chr4	+	0.67
NR_026680	Down	chr17	+	0.73
n332365	Down	chr6	−	0.71
n407274	Down	chr13	+	0.76
n336583	Down	chr8	−	0.68
NR_003135	Down	chr4	+	0.68
n335585	Down	chr6	−	0.62
TCONS_l2_00011393-XLOC_l2_006157	Down	chr17	−	0.75
n409338	Down	chr8	+	0.73
ENST00000437376	Down	chr10	+	0.67
n339699	Down	chr3	−	0.7
TCONS_00025100-XLOC_012113	Down	chr17	+	0.72
ENST00000410458	Down	chr22	+	0.63
n333458	Down	chr2	−	0.66
ENST00000440803	Down	chr10	−	0.75
n407951	Down	chr7	+	0.71
n340550	Down	chr5	+	0.66
NR_002953	Down	chrX	+	0.73
TCONS_l2_00031062-XLOC_l2_015962	Down	chrY	−	0.7
n385291	Down	chr7	−	0.71
n332754	Down	chr19	−	0.72
n335563	Down	chr5	−	0.68
n342719	Down	chr17	−	0.73
TCONS_00010981-XLOC_004915	Down	chr5	−	0.68
n405896	Down	chr4	+	0.7
n338326	Down	chr14	+	0.65
ENST00000539116	Down	chr12	+	0.72
n334838	Down	chr11	−	0.67
n339111	Down	chr16	−	0.64
TCONS_00011856-XLOC_005347	Down	chr6	+	0.62
n382989	Down	chr16	+	0.66
ENST00000408148	Down	chr4	−	0.38
ENST00000408407	Down	chr4	−	0.38
n408284	Down	chr11	−	0.66
n340108	Down	chr10	+	0.69
n408057	Down	chr21	−	0.73
n335665	Down	chr12	−	0.69
NR_003086	Down	chr10	+	0.74
n341449	Down	chr13	+	0.62
ENST00000560295	Down	chr8	−	0.71
n336585	Down	chr1	−	0.73
TCONS_l2_00014965-XLOC_l2_008329	Down	chr2	−	0.71
n381011	Down	chr12	−	0.69
ENST00000516262	Down	chr19	+	0.76
n337724	Down	chrX	−	0.62
ENST00000365465	Down	chr12	−	0.76
n339340	Down	chr3	−	0.76
n335618	Down	chr7	+	0.7
n383697	Down	chr2	+	0.73
NR_027451	Down	chr20	−	0.71
n386477	Down	chr8	−	0.61
n381720	Down	chr11	−	0.75
NR_003689	Down	chr5	+	0.75
n385130	Down	chr7	+	0.69
n338054	Down	chr15	+	0.73
n339273	Down	chr3	+	0.66
n339125	Down	chr16	+	0.75
TCONS_00007073-XLOC_002996	Down	chr3	+	0.71
n335629	Down	chr11	+	0.75
n338352	Down	chr14	+	0.71
TCONS_00020239-XLOC_009920	Down	chr12	+	0.72
n407424	Down	chr7	+	0.75
n410028	Down	chr11	+	0.73
n381557	Down	chr10	+	0.64
n409073	Down	chr11	+	0.76
n340600	Down	chr17	+	0.62
n384785	Down	chr5	−	0.67
n341705	Down	chr9	+	0.71
n346209	Down	chr1	−	0.69
n410553	Down	chr10	+	0.73
n337716	Down	chr15	+	0.72
ENST00000455464	Down	chr1	+	0.7
n407438	Down	chr14	+	0.71
n335719	Down	chr21	+	0.74
n341228	Down	chr7	+	0.69
ENST00000365473	Down	chr6	+	0.64
NR_026680	Down	chr17_ctg5_hap1	−	0.75
n339622	Down	chr3	+	0.67
ENST00000453508	Down	chrX	+	0.73
n332887	Down	chr12	−	0.74
n405865	Down	chr10	+	0.75
n342118	Down	chr6	+	0.61
n407887	Down	chr20	−	0.72
ENST00000412312	Down	chr2	−	0.73
n385370	Down	chr8	+	0.63
n407311	Down	chr1	−	0.74
TCONS_00030033-XLOC_014513	Down	chr7_gl000195_random	−	0.73
n342861	Down	chr9	−	0.75
ENST00000459514	Down	chr12	−	0.76
n337282	Down	chr11	+	0.68
NR_028379	Down	chrX	−	0.74
n345003	Down	chr8	−	0.69
n383521	Down	chr19	−	0.71
ENST00000424474	Down	chr1	+	0.75
n345156	Down	chr11	−	0.76
n332921	Down	chr5	−	0.72
n381233	Down	chr1	+	0.75
n332826	Down	chr9	−	0.76
n337780	Down	chrX	−	0.62
n340463	Down	chr17	−	0.74
n340776	Down	chr5	−	0.74
n332637	Down	chr4	−	0.68
n339112	Down	chr16	−	0.74
n333868	Down	chr17	+	0.74
n326264	Down	chr1	−	0.63
n384227	Down	chr3	−	0.72
n339624	Down	chr20	+	0.74
ENST00000516836	Down	chr15	+	0.73
ENST00000401195	Down	chr13	+	0.73
n337637	Down	chrX	+	0.71
n334856	Down	chr2	−	0.73
ENST00000458939	Down	chr5	+	0.68
n326195	Down	chr1	−	0.74
ENST00000533009	Down	chr11	−	0.7
n341273	Down	chr7	−	0.75
n335663	Down	chr3	+	0.73
n384440	Down	chr4	−	0.69
n339680	Down	chr3	−	0.55
n384435	Down	chr4	+	0.73
ENST00000459440	Down	chr7	+	0.72
n344955	Down	chr7	−	0.75
ENST00000408341	Down	chr3	−	0.76
n345418	Down	chr1	+	0.71
n338521	Down	chr14	−	0.74
n334200	Down	chr17	+	0.69
n382511	Down	chr15	−	0.72
n342042	Down	chr6	−	0.74
ENST00000458919	Down	chrX	+	0.74
ENST00000516343	Down	chr5	−	0.75
n335510	Down	chr20	−	0.75
ENST00000555853	Down	chr14	−	0.76
n338173	Down	chr22	+	0.72
ENST00000384483	Down	chr17	−	0.71
n386264	Down	chr2	+	0.67
n332849	Down	chr17	−	0.75
ENST00000390855	Down	chr1	−	0.75
ENST00000390928	Down	chr1	−	0.75
ENST00000391148	Down	chr7	+	0.75
n339455	Down	chr3	−	0.68
n341491	Down	chr8	+	0.65
NR_002936	Down	chr6	−	0.74
n341245	Down	chr7	−	0.61
ENST00000459168	Down	chr18	+	0.76
n339122	Down	chr2	−	0.71
n381271	Down	chr1	+	0.75
n332616	Down	chr10	+	0.71
n333506	Down	chr7	−	0.76
NR_003021	Down	chr14	−	0.73
n332612	Down	chr1	−	0.67
n384625	Down	chr5	−	0.74
ENST00000459540	Down	chr1	+	0.76
n385115	Down	chr7	−	0.74
NR_004398	Down	chr2	−	0.76
n339305	Down	chr16	+	0.75
n342319	Down	chr14	+	0.72
n408176	Down	chr2	−	0.76
n384541	Down	chr4	+	0.67
n382184	Down	chr14	+	0.74
TCONS_00015557-XLOC_007549	Down	chr9	+	0.76
n323966	Down	chr9	−	0.71
n339119	Down	chr2	+	0.76
ENST00000515929	Down	chr2	−	0.76
TCONS_00016238-XLOC_007621	Down	chr9	+	0.74
n337552	Down	chr2	−	0.75
n339967	Down	chr4	+	0.64

The primers used in the study were listed in Table 2 (Table 2).

**Table 2:** The primers used in the study.

18s	F:5′ CGGCTACCACATCCAAGGAA3′
	R :5′ GCTGGAATTACCGCGGCT3′
	
NONHSAT094064	F:5′ TCCGGAGCTGGTGCTGATAA3′
	R :5′ TGAGCAAGTGCTGAGGGTTTA3′
	
Ppargc1a	F:5′ CATGTGCAGCCAAGACTCTG3′
	R :5′ GTGAGGACCGCTAGCAAGTT3′
	
Ppargc1b	F:5′ TGAGGTGTTCGGTGAGATTG3′
	R :5′ CCATAGCTCAGGTGGAAGGA3′
	
Nrf1	F:5′ CTTCAGAACTGCCAACCACA3′
	R :5′ GCTTCTGCCAGTGATGCTAC3′
	
Erra	F:5′ GGAGGACGGCAGAAGTACAA3′
	R :5′ CAGGTTCAACAACCAGCAGA3′
	
Tfam	F:5′ CAAAAAGACCTCGTTCAGCA3′
	R :5′ CTTCAGCCATCTGCTCTTCC3′
	
ME1	F：5′ CTGCTGACACGGAACCCTC3′
	R: 5′ GATCTCCTGACTGTTGAAGGAAG3′
	
ALG10B	F：5′ GCATCCTTTGCCTTCCGTGG3′
	R: 5′ CTTGAGGCAGCCTTGTTTCTG3′

**Fig. 1:**
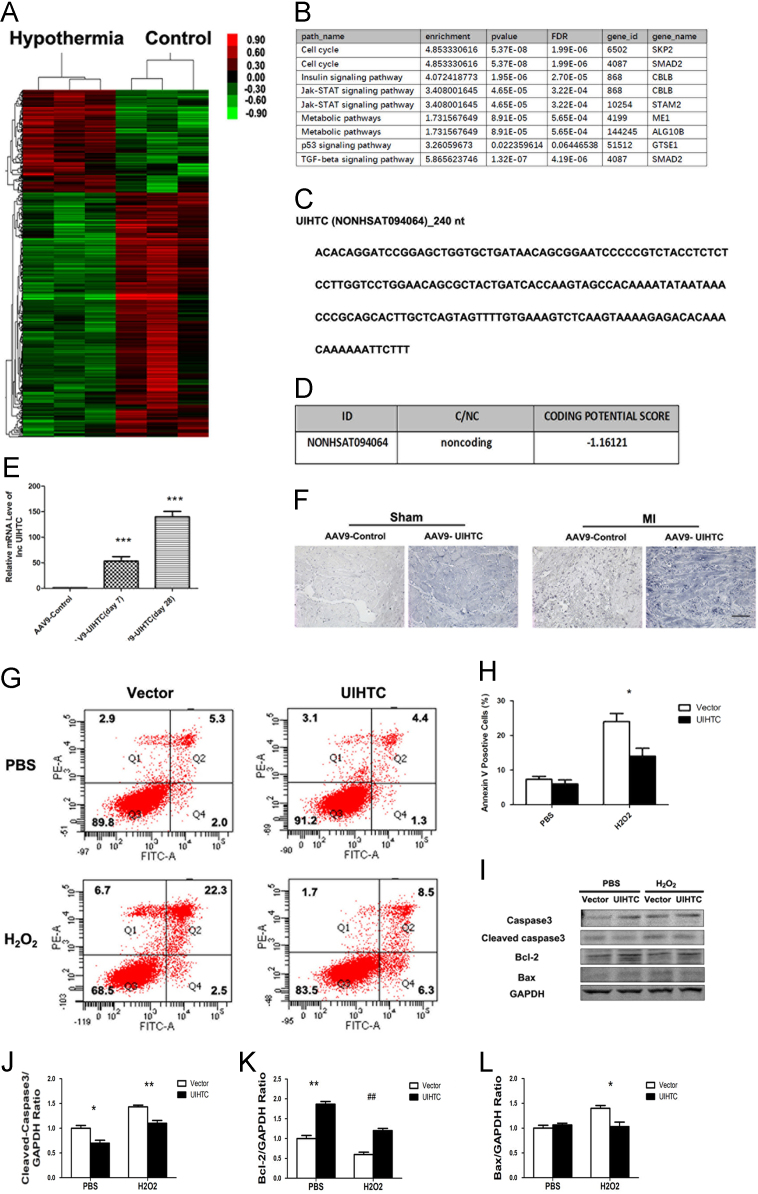
UIHTC protects against MI **A**, Hierarchical clustering based on levels of LncRNAs in AC16 cell treated with hypothermia or not. **B**, KEGG Pathway analysis of LncRNA-UIHTC. **C**, The full sequence of LncRNA-UIHTC. **D**, The Coding Potential Calculator result indicates that LncRNA-UIHTC is no-coding RNA. **E**, Real-time PCR quantification of UIHTC expression in indicated days. ***, *p* < 0.001 vs AAV9-Control. **F**, in situ hybridization showing staining of UIHTC in cardiac myocytes (scale bar represents 100 µm). **G, H**, Apoptosis rate of AC16 cells was decreased in UIHTC transfected cells. *, *p* < 0.05 vs Vector H_2_O_2_. **I**, Caspase 3, Bcl-2 and Bax were determined by Western blot in indicated cells. **J**, Quantitation of Cleaved casepase3 and GAPDH. *, *p* < 0.05 vs Vector PBS; **, *p <* 0.01 vs Vector H_2_O_2_. **K**, Quantitation of Bcl-2 and GAPDH. **, *p <* 0.01 vs Vector PBS; ##, *p <* 0.01 vs Vector H_2_O_2_. **L**, Quantitation of Bax and GAPDH. *, *p <* 0.05 vs Vector H_2_O_2_.

Hierarchical clustering based on levels of LncRNAs in AC16 cell treated with hypothermia (Fig. 1A). The KEGG Pathway analysis of LncRNA-UIHTC (lncRNA upregulated in hypothermia treated cardiomyocyte, NONHSAT094064) was shown in (Fig. 1B). The full sequence of LncRNA-UIHTC has been shown in (Fig. 1C). Moreover, the Coding Potential Calculator result indicates that LncRNA-UIHTC is no-coding RNA (Fig. 1D). Then, we overexpression of UIHTC by AAV9 in rat and found that UIHTC was successfully overexpressed in rat after MI 28 days (Fig. 1E and F). Next, we overexpressed UIHTC in AC16 cells and found that UIHTC inhibited H_2_O_2_ induced apoptosis (Fig. 1G–I).

**Fig. 2:**
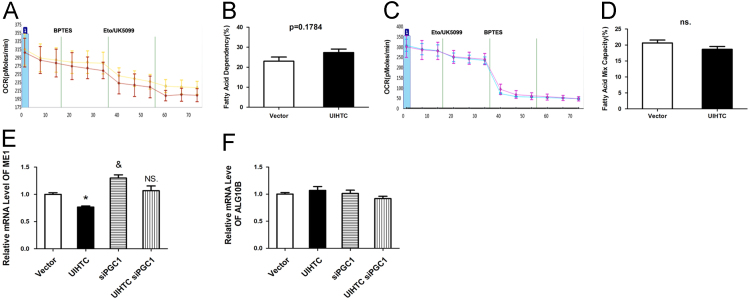
UIHTC protected cardiomyocytes through enhancing mitochondrial function. **A–D**, Figure shows the fatty acid dependency and capacity of control and overexpressed UIHTC cells. **E**, Real-time PCR quantification of ME1 expression. *, *p* < 0.05 vs Vector; &, *p* < 0.05 vs Vector; ns, *p* > 0.05 vs Vector. **F**, Real-time PCR quantification of ME1 expression.

UIHTC expressed AC16 exhibited no significantly capacity to use and increase oxidation of glucose when trying to compensate for BPTES- and etomoxir-induced inhibition of alternative fuel pathways (i.e., glutamine oxidation and long chain fatty acid oxidation, respectively) (Fig. 2A–D).

## 2 Materials and methods

### 2.1 Human specimens

All the fresh human tissue specimens used in this study were obtained from patients who underwent heart transplantation for heart failure at Department of Cardiovascular Surgery, General Hospital of Shenyang Military Area Command in Shenyang, Liaoning, China. The procedure of human sample collection and analysis was approved by the Ethics Committee of General Hospital of Shenyang Military Area Command.

### 2.2 Cells culture and microarray analysis

Cell line of human adult ventricular cardiomyocytes (AC16) and microarray data has been described before [1]. The microarray data have been deposited in NCBI Gene Expression Omnibus and are accessible through GEO Series accession number GSE71361.

### 2.3 Gene silencing and plasmid transfection

Synthetic small interfering RNA (siRNA; scrambled, PGC1α and UIHTC -targeting siRNA) were purchased from Genepharma Co. (Shanghai, China). The sequences of siRNA against PGC1α are shown as follow: siPGC1α #1 forward: GCCAAACCAACAACUUUAUUU; reverse: AUAAAGUUGUUGGUUUGGCUU. The sequences of siRNA against UIHTC are shown as follow: si UIHTC #1 forward: GUGCUGAUAACAGCGGAAUTT; reverse: AUUCCGCUGUUAUCAGCACTT; si UIHTC #2 forward: CGCUACUGAUCACCAAGUATT; reverse: UACUUGGUGAUCAGUAGCGTT. The transfection of siRNAs was performed according to the manufacturers’ instructions. For the plasmid construction, the full-length of UIHTC was subcloned into an XhoI/EcoRI site of a transposon-based pEX-2 vector.

### 2.4 in situ hybridization

For in situ hybridization, the probe used for detecting UIHTC-labeled digoxin was designed and synthesized by Takara (Dalian, China). The probe sequence was designed as 5DigN/TACTTGGTGATCAGTAGCGCT/3Dig_N. Hybridization was performed using the in situ hybridization Kit (Boster Bio-Engineering Company, Wuhan, China) according to the manufacturer's instructions.

### 2.5 Real-time PCR analysis

The RNA of each sample was extracted from the infarction and border zone. Real-time PCR was performed using a SYBR Green PCR Kit (Applied Biosystems) and an ABI 7900HT Fast Real-Time PCR System (Applied Biosystems). The sequences of primers used were listed in Table 2.

### 2.6 Western blot analysis

The protein of each sample was extracted from the infarction and border zone. Western blot analysis was carried out as described previously [2]. The nitrocellulose filter membranes were incubated overnight at 4 °C with diluted antibodies against Caspase 3 (1:1000, CST), Bcl-2 (1:200, Santa Cruz), Bax (1:200, Santa Cruz), or PGC1α (1:200, Santa Cruz).

### 2.7 Masson trichrome staining

Masson trichrome staining was carried out as described previously [3]. Briefly, Paraffin-embedded tissue samples were sectioned (3 µm) and Masson's trichrome staining (Baso Diagnostics Inc. Zhuhai, China) was performed following the manufacturer's instruction.

#### 2.8 Detection of myocardial cell apoptosis of myocardial cell apoptosis

After heart tissue section, myocardial apoptosis was detected using terminal deoxynucleotidyltransferase-mediated dUTP nick end labeling (TUNEL) (Roche, Basel, Switzerland).

### 2.9 AAV9-mediated gene expression

The sequence of human UIHTC was cloned into the p3×Flag CMV vector. AAV9-EGFP and AAV9-EGFP- UIHTC were prepared by Hanbio Biotechnology Co., Ltd. (Shanghai, China).

### 2.10 Myocardial infarction model and experimental design

MI rat model was established as described previously [4]. The rats were anesthetized with 1% sodium pentobarbital (50 mg/kg). Myocardial ischemia surgery was performed by exposing the heart with a left thoracic incision. A silk ligature was placed around left anterior descending coronary artery. Immediately after ligation, 30 μl of recombinant AAV9 vector (AAV9-UIHTC or AAV9-control; 1 × 10^11^ vg per animal) was injected around the infarction region.

### 2.11 Echocardiography

Structural and functional parameters were assessed in sedated (Isoflurane 1.0%) rats by echocardiography (Visualsonics VEVO 2100). Under anesthesia, the chest was shaved, and two-dimensional echocardiography was performed using our echocardiographic. M-mode and B-mode images were collected at baseline, as well as at 2, and 4 weeks. The LV ejection fraction (%EF) and fractional shortening (%FS) were automatically calculated by the echocardiographic system.

### 2.12 Measurement of endogenous ROS level

The intracellular ROS levels were detected by labeling 2 × 105 AC16 cells with redox-sensitive probes CellRox (5 μM) (Life Technologies) for 30 min at 37 °C. Then the cells were washed twice and resuspended in 0.2 ml PBS. Fluorescence of labeled cells was analyzed by flow cytometry.

### 2.13 ATP measurement

The ATP content in cells was determined using a Luciferase-based Bioluminescence Assay Kit (Beyotime, Haimen, China) following the manufacturer's instruction. The protein of each sample was extracted from the infarction and border zone.

### 2.14 Cellular GSH, NADPH assays

The intracellular NADP/NADPH ratio were determined using a NADP/NADPH Quantitation Colorimetric Kit (Biovision) according to the manufacturer instructions. For measurement of GSH content, a Glutathione (GSH) Assay Kit (Beyotime, Haimen, China) was used as recommended instructions. The protein of each sample was extracted from the infarction and border zone.

### 2.15 JC-1 assay

AC16 cells mitochondrial membrane potential was measured by mitochondrial membrane potential detection kit (JC-1) (Beyotime, Haimen, China).

### 2.16 Metabolic assays

Oxygen consumption rate (OCR) and Extracellular acidification rate (ECAR) were evaluated using the Seahorse XF96 extracellular flux analyzer. One hour before XF assay, AC16 cells (50,000/well) were equilibrated with unbuffered DMEM and maintained in 37 °C for PH stabilization. Analyses were performed both at basal conditions and after injection of OLI (2 µM), FCCP (0,25 mM), Antimycin A (0.5 mM) at indicated time points.

Metabolic dependence was analyzed by XFp Mito Fuel Flex Test kit. Analyses were performed both at basal conditions and after injection of BPTES (3 µM), ETO (4 mM) and UK5099 (2 µM) at indicated time points according to manufacturer's instructions.

## 3 Statistical analysis

RVM t-test was applied to filter the differentially expressed lncRNAs for the control and hypothermia treated group because the RVM *t*-test can raise degrees of freedom effectively in the cases of small samples. After the significant analysis and FDR analysis, we selected the differentially expressed genes according to the *p*-Value threshold. *p* Value < 0.05 was considered as significant difference. Results are presented as mean ± SD. Unpaired two-tailed Student's *t*-test or one-way ANOVA analyzed all the data. Statistical significance was set at *p* < 0.05. Statistical analysis was carried out using Prism Software (GraphPad).
