# Supplementary material for: Data on long noncoding RNA upregulated in hypothermia treated cardiomyocytes protects against myocardial infarction through improving mitochondrial function
Source: Data Brief. 2018 Feb 5;17:610–25. doi: 10.1016/j.dib.2018.01.052 (PMC5852326; doi:10.1016/j.dib.2018.01.052)
Supplement: Supplementary file 1 — Supplementary material [file mmc1.docx]

**Disclosure**

None.
